# Centrality of the COVID-19 outbreak and subsequent post-traumatic growth in nurses: Exploring the moderating role of social connectedness

**DOI:** 10.1177/10519815251334103

**Published:** 2025-04-17

**Authors:** Catarina Vitorino, Maria Cristina Canavarro, Carlos Carona

**Affiliations:** 1 Center for Research in Neuropsychology and Cognitive-Behavioral Intervention, Faculty of Psychology and Educational Sciences, University of Coimbra, Coimbra, Portugal

**Keywords:** nurses, COVID-19, trauma, adaptation outcomes, resilience

## Abstract

**Background:**

In the aftermath of the COVID-19 pandemic, nurses may report positive transformations due to the struggles faced during the outbreak, but the factors explaining this phenomenon remain understudied.

**Objective:**

Considering the crucial role played by social ties and support to overcome adversity, the present research aims to explore social connectedness as a moderator between centrality of the COVID-19 outbreak and subsequent post-traumatic growth (PTG) in nurses. Specifically, the study sought to examine whether the longitudinal association between centrality of the COVID-19 outbreak and subsequent nurses’ PTG differed according to the levels of social connectedness.

**Methods:**

The global sample was composed of 180 nurses working in Portuguese healthcare institutions who answered online self-report surveys at two time points (baseline [T1] and follow-up at 6 months [T2]). Using self-report questionnaires, measures of centrality of the COVID-19 pandemic outbreak and social connectedness were administered at the baseline, and PTG reports were obtained at the six-month follow-up. Attrition rate from T1 to T2 was 73%.

**Results:**

Results showed that social connectedness was a significant moderator in the relationship between centrality of the COVID-19 outbreak and PTG, with that association being stronger as the levels of social connectedness increased.

**Conclusions:**

These findings shed light on the importance of social connectedness in fostering PTG among nurses after the COVID-19 outbreak. Psychological interventions aimed at enhancing nurses’ constructive adjustment outcomes should acknowledge the occurrence of resilience and promote feelings of social connectedness and safeness to amplify potentially adaptive effects of the transformative nature of the pandemic.

## Introduction

A growing body of empirical studies has suggested that nurses may describe, not only negative effects but also positive transformations in the aftermath of the COVID-19 pandemic outbreak.^1,2^ Still, the specific pathways explaining the phenomenon of post-traumatic growth (PTG) remain unclear in the context of this global health crisis, especially for nursing professionals.

PTG reflects positive psychological and personal changes that may emerge from the struggle triggered by major life challenges, involving aspects such as relating to others, new possibilities, personal strength, spiritual change, and appreciation of life.^3,4^ According to the revised model of PTG,^5^ individual (e.g. assumptive beliefs) and environmental (e.g. social support) factors interact and foster the need to re-evaluate and rebuild pre-trauma appraisals, thus creating opportunities for new life meanings and motivations. Some factors that may promote PTG include sharing negative emotions, cognitive processing or rumination, positive coping strategies (e.g. positive reappraisal), personality traits (e.g. agreeableness), experiencing multiple sources of trauma, event centrality, resilience, and growth actions.^6^

Personal growth often entails disruptive events that challenge individuals’ representations of the self, others, the world, and the future, thus evoking the centrality of the event.^7^ Event centrality is the subjective perception of highly stressful situations as life-changing, according to the effects on one's identity, everyday inferences and life story.^8^ This extreme disruption of one's goals and core beliefs paves the way for cognitive processing of the event, which is a key factor in the psychological impact of traumatic experiences.^5^ The predictive role of event centrality in the development of PTG has been largely documented.^9,10^ However, most studies are cross-sectional, and the existing literature on the longitudinal relationship between these variables is scarce and did not produce conclusive results.^11–13^

Even though traumatic events may be perceived as central to one's life, different adjustment outcomes can arise from adversity. Social support is emphasized in the revised model of PTG as one of the most significant factors for growth, by stimulating the process of meaning-making of the event and the emotions, increasing internal resources, and decreasing the impact of stress on psychological health.^5^ Findings from a recent meta-analysis corroborated the beneficial effects of social support on self-reported PTG, in both cross-sectional and longitudinal studies, which underlines the importance of promoting this resource to ensure constructive outcomes in the aftermath of trauma.^14^

Two models are commonly used to explain the mechanisms behind the moderating effect of social support on the ability to cope with a traumatic event. First, the “direct or main effects” model suggests that social support can benefit well-being, regardless of stress levels. According to the “buffer” model, social support can interact with stress and potentially reduce, inhibit, or counteract the negative effects of stressful events.^15^ Both perspectives identify the conditions under which the effects occur, but the “buffer” model is the one highlighted in the current research.

Multiple pathways can explain PTG based on whether a person perceives their relationships with others as warm, reassuring, and safe. Therefore, research on the role of interpersonal variables has shown the need to focus on the psychological and affective dimension of social ties, rather than the support itself.^16^ Within the context of the COVID-19 pandemic, a study across 21 countries worldwide confirmed the key role of social connectedness in the development of PTG.^17^ Feelings of social connectedness and safeness have been associated with the activation of the soothing-affiliation system, which enables social exploration, tones down threat responses, and increases positive mental health outcomes.^18^

### The current study

The context of the COVID-19 aftermath can be a unique opportunity to better understand the phenomenon of growth.^19,20^ Due to additional challenges both at work and at home (e.g. scarcity of resources, work overload, managing family responsibilities), the process of transformation of suffering into growth is of paramount importance to understand and replicate in future crises.

The main goal of the present study was twofold: first, to determine the predictive value of the centrality of the COVID-19 outbreak on nurses’ subsequent PTG; and second, to examine the moderating role of social connectedness on that link.

## Methods

### Participants

The present study comprises a retrospective, longitudinal design with two points of data collection: baseline (T1) and follow-up at 6 months (T2). The first assessment (T1) was conducted between September 2021 and May 2022 to explore nurses’ perceived centrality of the COVID-19 pandemic outbreak and appraisals of social connectedness. The second assessment (T2) was carried out between May 2022 and December 2022, and it investigated nurses’ self-reported PTG.

To participate in the study, individuals only had to fulfill one inclusion criterion, which was being a nurse working in a Portuguese hospital or any other healthcare institution during the time of the COVID-19 outbreak. Of the 672 nurses assessed at baseline, 180 completed the follow-up assessment (26.8%). Thus, the attrition rate from T1 to T2 was 73%, which is in line with previous reports on postdisaster longitudinal research.^21,22^ When comparing sociodemographic and clinical variables between nurses who did versus did not participate in the follow-up, the only variables that showed statistically significant differences between both groups were marital status, being infected with the coronavirus, and being isolated from significant others.

### Procedure

All procedures complied with the Declaration of Helsinki and its later amendments for research with humans.^23^ The study had the approval of the Ethics Committee of the Faculty of Psychology and Educational Sciences of the University of Coimbra.

Data were collected through the non-probabilistic sampling methods of snowball and convenience sampling. Institutional email lists and social and traditional media platforms were used to share the assessment protocol by applying unpaid cross-posting, paid advertisements, and booster campaigns. In addition, the dissemination of the assessment protocol was facilitated by the Portuguese Order of Nurses, Portuguese Nurses Unions and the Nursing Schools of Coimbra and Lisbon, which agreed to collaborate with the project.

A web-based platform (LimeSurvey^®^) was used to administer the Portuguese validated versions of the self-report measures. Data cleaning techniques were embedded into survey design to prevent the recruitment of biased, duplicate, or fake respondents.^24^

For the baseline assessment, participants were informed about the study aims, inclusion criteria, and conditions in the first page of the questionnaire (i.e. steps of the data collection, researchers’ roles, and the voluntary, anonymous, and confidential nature of the participation), to which they had to consent before starting the survey. At the beginning of the survey, an identification code (the first three letters of their name and the last three digits of their mobile phone number) was devised to protect the anonymity of the participants. Nurses were also requested to give an e-mail contact in order to be invited to voluntarily participate again in the study at the follow-up assessment.

After 6 months of their participation, nurses automatically received an invitation by e-mail to answer the assessment protocol corresponding to the follow-up.

### Measures

#### Sociodemographic and clinical information

For the first moment of assessment of the present study, a sociodemographic and clinical survey was created to collect information about nurses’ sociodemographic (e.g. age, gender) and health-related data (e.g. psychological/psychiatric treatment history). Data on the level of exposure to the virus of SARS-CoV-2 (e.g. isolated from significant others), as well as the characteristics of the working context during the pandemic outbreak (e.g. whether they worked in a COVID-19 unit) were also collected.

#### Event centrality

The Centrality of Event Scale^8,25^ evaluates the extent to which a memory of an extremely traumatic event (in this case, nurses were asked to: “Think about the COVID-19 outbreak”) is appraised as pivotal to one's self-knowledge and life narrative. This self-report measure has 20 items assessed on a 5-point Likert scale, ranging between 1 (*totally disagree*) and 5 (*totally agree*). The total score of the scale corresponds to the sum of all items, in which higher scores are associated with a greater event centrality. In the present research, the scale revealed an internal consistency of ω = .94; α = .94. s

#### Social connectedness

The Social Safeness and Pleasure Scale^18,26^ assesses social connectedness and the level of safeness, warmth, and reassurance individuals feel in their interpersonal connections. It comprises 11 items, rated by nurses on a 5-point Likert scale from 1 (*almost never*) to 5 (*almost all the time*), according to the frequency they experience feelings of connection with others. Higher scores indicate greater levels of social connectedness. In the present study, internal consistency values were ω = .94; α = .94.

#### Post-traumatic growth

The Post-traumatic Growth Inventory – Short Form,^27,28^ a short version of the Posttraumatic Growth Inventory,^3^ investigates the subjective positive psychological changes in the aftermath of highly traumatic events (here, nurses were asked to: “Think about the COVID-19 outbreak”). It is composed of 10 items rated on a 6-point Likert scale, ranging between 0 (*I did not experience this change as a result of my crisis*) and 5 (*I experienced this change to a very great degree as a result of my crisis*). By summing up all items is calculated the total score. Higher scores indicate higher levels of PTG. In the present study, internal consistency was ω = .90; α = .92.

### Data analysis

Statistical Package for the Social Sciences (SPSS, version 27.0; IBM SPSS, Chicago, IL, USA) and the PROCESS computation tool (version 4.2 for SPSS)^29^ were used to conduct statistical analyses. Based on a priori power analysis (G*Power),^30^ a minimum of 107 individuals was required to detect medium-to-large effects in planned statistics (i.e. multiple linear regression).

In order to determine the internal consistency of the scales included in the present study, Cronbach's alphas and McDonald's omega were calculated, considering α ≥ .80 as optimal.^31,32^ The Kolmogorov-Smirnov test was used to assess normality of the scales, supporting the assumption that the data follows a normal distribution. Pearson's bivariate correlation coefficients were performed to calculate associations between sociodemographic/clinical indices and variables of interest, adopting the following parameters: *r *≤ .29 (weak); .30 ≤ *r *≤ .49 (moderate); *r *≥ .50 (strong).^33^

A simple moderation model (Model 1) was adopted to explore the moderating effect of social connectedness between centrality of COVID-19 outbreak and post-traumatic growth.^29^ Before the analysis, all variables were mean centered for products calculation. The statistical significance of the main and interaction effects was tested using a bootstrapping procedure with 10,000 samples, which generated 90% bias-corrected and accelerated confidence intervals (90% BCaCIs).^34^ When the lower and upper confidence interval did not contain the value of zero, the main and interaction effects were considered significant. PROCESS-generated data were used to facilitate post-hoc graphical depictions of conditional effects of event centrality on PTG for low (−1SD below the mean), medium (mean), and high (+1SD above the mean) levels of social connectedness. The following covariates were included in the tested model taking in consideration the significant correlations observed between the dependent variable and socio-professional characteristics^34^: age (*r *= −.28, *p *< .001) and years of professional experience (*r *= −.26, *p *< .001). Effect sizes of main and interaction effects were based on the values of *R*^2^, which were then classified as small (*R*^2 ^≥ .02), medium (*R*^2 ^≥ .13) and large (*R*^2 ^≥ .26).^35^

## Results

### Sociodemographic and clinical characteristics of the sample

Table 1 illustrates the sociodemographic and clinical information of the sample, composed by 180 nurses (age: *M *= 38.9; *SD *= 9.36). The majority identified as a woman (*n *= 164, 91.1%), were married (*n *= 97, 53.9%), and lived in an urban area (*n *= 138, 76.7%). The mean years of professional experience was 15.7 (*SD *= 9.34) and 52.8% (*n *= 95) of the nurses worked at a COVID-19-specific unit during the pandemic.

**Table 1.** Sociodemographic and clinical characteristics of the sample.

	Nurses	
	*N*	*%*
Gender		
Woman	164	91.1
Man	15	8.3
Non-binary	<5	<2.8
Marital status		
Single	73	40.6
Married	97	53.9
Divorced	8	4.4
Widow	<5	<2.8
Residential area		
Urban	138	76.7
Rural	42	23.3
Psychological/psychiatric treatment historyª	86	47.8
Isolated from significant othersª	61	33.9
Risk group for COVID-19ª	48	26.7
Infected with the coronavirusª	51	28.3
Worked at a COVID-19 unitª	95	52.8

Nurses were on average 38.9 years old (*SD *= 9.36), with 15.7 years of professional experience (*SD *= 9.34).

ª Reflects the number and percentage of participants answering “yes” to this question.

The only statistically significant correlation between the clinical indices and the variables of interest was working at a COVID-19 unit. This association was negative with event centrality (*r *= −.275), and positive with social connectedness (*r *= .204).

### Correlations between pandemic centrality, social connectedness, and PTG

As illustrated in Table 2, event centrality was not statistically correlated with social connectedness. The correlation between event centrality and PTG was positive and moderate, while social connectedness and PTG were weakly and positively correlated.

**Table 2.** Matrix of inter-correlations among study variables.

	*M*	*SD*	IQR	*r*	
				1.	2.
1. Event centrality	61.22	16.85	25	-	-
2. Social connectedness	39.88	9.20	11	−.07	-
3. Post-traumatic growth	17.16	10.62	16	.36**	.13*

IQR: Inter-quartile range.

* *p *< .10, ** *p *< .05.

### The moderating role of social connectedness between the association of event centrality and PTG

The main effects of event centrality (*b *= .21, *SE *= .04, 90% CI [.1411, .2862]) and social connectedness (*b *= .23, *SE *= .08, 90% CI [.0984, .3584]) on post-traumatic growth were statistically significant. The interaction effect was also significant (*b *= .01, *SE *= .00, 90% CI [.0024, .0167]), which means that social connectedness moderated the relationship between event centrality and post-traumatic growth. The model explained 24% (*R^2 ^*= .24, F(5, 174) = 10.75, *p *< .001) of the variance of post-traumatic growth, with an overall medium effect size. Results from the moderation analysis are detailed in Table 3.

**Table 3.** Main and interaction (moderating) effects of event centrality and social connectedness on post-traumatic growth.

	*B*	*SE*	*t*	*p*
*R*^2 ^= . 24				
Constant	2.15	6.61	.33	.75
Event centrality	.21	.04	4.87	.00
Social connectedness	.23	.08	2.91	.00
Event centrality × Social connectedness	.01	.00	2.21	.03

**Figure 1.**
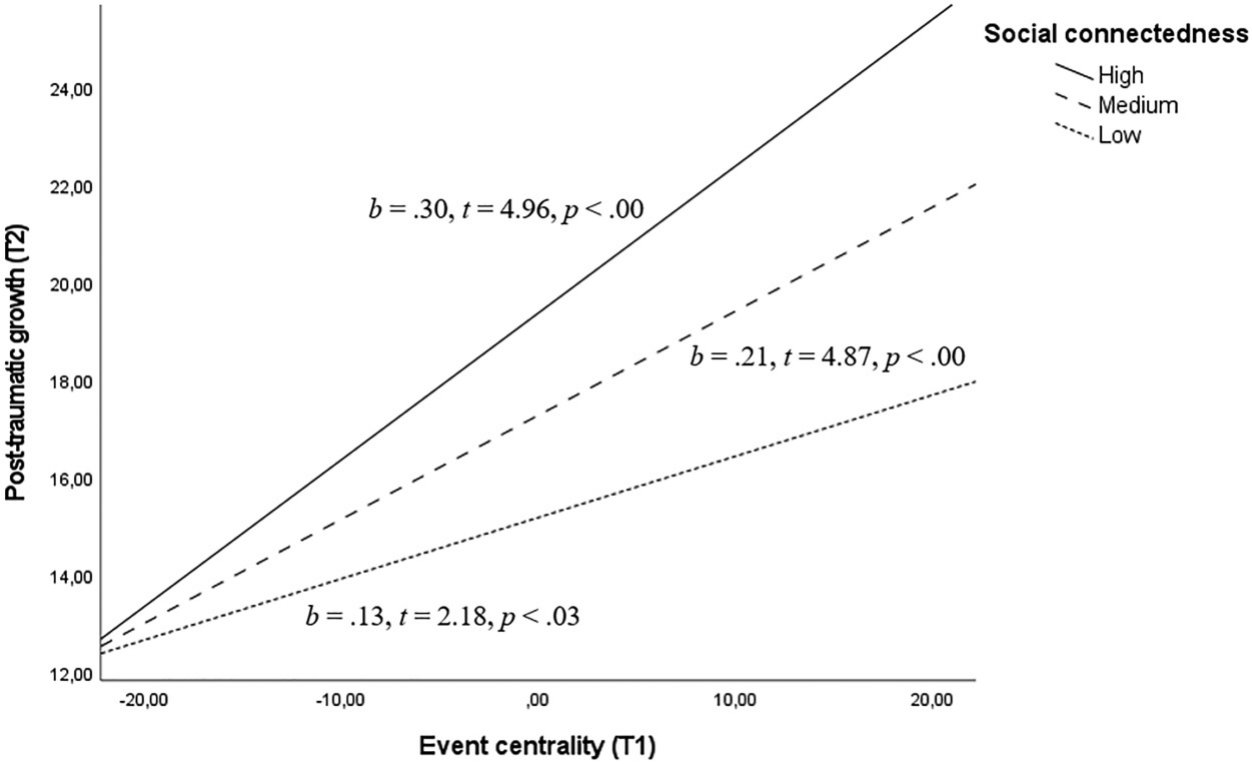
The moderating role of social connectedness on the association between the centrality of the COVID-19 pandemic outbreak and nurses’ subsequent PTG.

Conditional effects of event centrality on PTG, as depicted in Figure 1, were significant for all levels of social connectedness, but that association was progressively stronger as the scores of the moderating variable increased.

## Discussion

The COVID-19 pandemic was considered a mass traumatic event, with worldwide deleterious effects for nurses, but exposure to COVID-19-related stressors might also have changed them positively, by providing the opportunity to find new meanings and life purposes.^36^ This study sought to address a major gap regarding the role of social connectedness on the complex experience of PTG, particularly for nurses in the aftermath of the COVID-19 pandemic outbreak.

The main findings of this study can be summarized as follows: first, centrality of the pandemic outbreak and social connectedness had significant direct effects on PTG; second, the link between centrality of the pandemic outbreak and PTG was moderated by social connectedness; specifically, the moderating effects of social connectedness were significant, with the association between pandemic centrality and subsequent PTG being gradually stronger concurrently with the increasing scores of the moderator.

First, there was a positive direct link between pandemic centrality and self-reported PTG, which confirms that the development of psychological transformations in the aftermath of a traumatic event is strongly related to the subjective experience of the situation. Nurses for whom the pandemic outbreak occupied a central personal memory tended to report higher levels of subsequent PTG. Considering the major challenges faced by nurses, as well as the extreme disruption of their routines and core beliefs, the COVID-19 pandemic was considered life-changing. As such, the transformative process of reexamination of core beliefs seemed to facilitate the incorporation of this health crisis in the direction of growth. Several cross-sectional studies with other populations who had faced different adversities have consistently shown that event centrality was a strong predictive factor of self-reported PTG,^9,10^ but the obtained results in our study ascertain the longitudinal predictive effects of event centrality.

Moreover, it was found that social connectedness was a significant moderator between COVID-19 pandemic centrality and self-reported PTG. This finding suggests that appraising the pandemic as self-defining led to more positive psychological and personal transformations 6 months after the COVID-19 outbreak, but that association was systematically stronger when relationships with others were experienced as safer. Additionally, the strength of that moderating effect appeared more evident as the levels of event centrality increased. As such, nurses who felt connectedness with others during the COVID-19 outbreak tended to perceive the pandemic as a central component of their (self-)narrative, which may have contributed to the development of enhanced psychological growth. In addition to the well-documented role played by family and friends in adaptation to adversity,^2,37^ workplace interpersonal dynamics may have been crucial for the ability to cope with this global health crisis.^38^ Supportive peer relationships may have fostered feelings of confidence in oneself and others, the capacity to explore the environment, and a sense of common humanity and shared purpose that can be a catalyst to find a positive and adaptive meaning for the pandemic.

The results also indicate that nurses who did versus those who did not participate in the follow-up assessment show similar sociodemographic and clinical characteristics, excluding marital status, being infected with the coronavirus, and being isolated from significant others. It is also worth noting that half of the nurses worked at a COVID-19-specific unit during the pandemic, and reported a history of psychological/psychiatric problems, which may suggest an increased vulnerability to burnout and other mental health disorders. The fact that they asked for professional help could also be attributed to heightened awareness of mental health issues.

Finally, the association between having worked at a COVID-19 was negative with event centrality (r = −.275), which means that those who worked at a COVID-19 unit reported higher levels of event centrality. This is to say that the direct exposure to COVID-19-related stressors and infected patients was appraised by nurses as a central experience to their identity and life narrative. On the other hand, being in contact with the coronavirus in the working context was positively related to social connectedness. As such, nurses working in COVID-19 units felt their social connections were less safe, warm and soothing.

These results shed light on significant implications for clinical practice and health policy. Developing empowered healthcare workers is essential to provide quality healthcare services while retaining dignity for patients and themselves.^39^ Acknowledging that growth is a complex phenomenon, involving both positive and negative emotions, is vital to conceptualize the process of adaptation through a comprehensive and clinically informative perspective. Since PTG is characterized by a period of suffering, a primary approach should concomitantly address both psychological distress and personal growth. Psychological interventions should be aimed at building new narratives and life meanings in the aftermath of challenging times, such as the pandemic, that are in line with a person's values, goals, and core beliefs. Accordingly, healthcare institutions should also prioritize the adoption of proactive measures to create a safe working place environment where professionals can develop nurturing relationships and feel emotionally supported and connected. Additionally, by working through compassionate lens, nurses may experience changes in self-perception and relationships with others and the world, by cultivating their capacities for compassion mentalities and caring motives. Feelings of social safeness can then facilitate constructive memories, meanings, and motivations that derive psychological growth in the aftermath of such traumatic events.

Despite the contributions of the present study, some limitations should be considered. First, the validity of self-perceived PTG has been questioned by some authors due to the suspected overestimation of self-reports, depending on whether participants answered the questions about a particular event or generally about recent changes in their lives. However, research comparing both approaches shows that attributing growth to specific stressors may actually lead individuals to underestimate their growth, contradicting the notions that self-reports of PTG may be positively biased.^40^ Even though there are studies that underline the importance and validity of relying on nurses’ own voices,^20^ future research should use more sensitive designs and consider multiple informants to comprehensively investigate such adaptation outcomes. Second, this study relied exclusively on online self-report questionnaires, thus excluding participants for whom the internet is not accessible or who do not have interest on social media at all. Thus, data collection in future studies should ideally supplement online recruitment with paper-and-pencil questionnaires. Given the volatility of pandemic peaks, the time of data collection can influence the obtained results, and may not be representative of other periods of this crisis. Nurses were invited to participate on the web through social and traditional media platforms using unpaid cross-posting, paid advertisements, and booster campaigns, which may also increase the risk for self-selection bias (i.e. nurses with greater interest in the study or experiencing higher distress may have been prone to participate), thus not ensuring the representativity of all nurses in the resultant sample. Furthermore, the literature has highlighted some inconsistencies about the longitudinal effects of event centrality,^12^ which may justify the need to develop studies with additional moments of prospective assessment. Although an increased dropout rate may be expected, 73% is notably high. However, healthcare workers face exceptionally demanding work environments, coupled with the number of study invitations received, which can contribute to participant attrition.^41^ Moreover, the traumatic experiences endured during this period may evoke avoidance behaviors, hindering recall and willingness to engage with research. Future studies may benefit from offering compensation or implementing intervention programs to enhance participant engagement. Finally, the specific sample in which data were collected may limit the generalizability of findings to other vulnerable groups or cultural contexts, thus reinforcing the need to replicate this research for different populations.

## Conclusion

The results underscore the importance of subjective experiences and interpersonal relationships in fostering PTG following a traumatic event. Healthcare institutions should prioritize creating supportive work environments that promote social connectedness and emotional well-being among nurses. Additionally, psychological interventions aimed at building new narratives and life meanings can help individuals navigate the challenges of post-traumatic growth.
